# Synergistic effect of eicosapentaenoic acid-enriched phospholipids and sea cucumber saponin on orotic acid-induced non-alcoholic fatty liver disease in rats

**DOI:** 10.1098/rsos.172182

**Published:** 2018-07-11

**Authors:** Ying Guo, Xiuqing Han, Hongxia Che, Zhaojie Li, Ping Dong, Changhu Xue, Tiantian Zhang, Yuming Wang

**Affiliations:** 1 College of Food Science and Engineering, Ocean University of China, No. 5 Yushan Road, Qingdao, Shandong Province 266003, People's Republic of China; 2 Laboratory for Marine Drugs and Bioproducts of Qingdao National Laboratory Marine Science and Technology, Qingdao, Shandong Province, People's Republic of China

**Keywords:** non-alcoholic fatty liver disease, EPA-enriched phospholipids, sea cucumber saponin, synergistic effect, lipid metabolism

## Abstract

Non-alcoholic fatty liver disease (NAFLD) is becoming an increasingly prevalent chronic liver disease all over the world. The present study was undertaken to explore the synergistic effects of sea cucumber saponins (SCS) and eicosapentaenoic acid-enriched phospholipids (EPA-PL) at ratios of 0.5 : 0.5 and 1 : 1 on NAFLD and demonstrate possible protective mechanisms. It was found that the combination of EPA-PL and SCS at half dose exhibited better effects than EPA-PL or SCS alone and the combination of EPA-PL and SCS at full dose in alleviating orotic acid (OA)-induced symptoms including growth parameters, serum parameters and liver function. Further evaluation of the mechanism illustrated that EPA-PL and SCS combination at the ratio of 0.5 : 0.5 could markedly reduce the mRNA expressions of fatty acid synthase, acetyl-CoA carboxylase, glucose-6-phosphate dehydrogenase and malic enzyme genes and significantly increase expression of genes relevant to fatty acid β-oxidation including peroxisome proliferator-activated receptor and its target genes (CPT1, CPT2 and ACOX1), suggesting that the protection of the EPA-PL and SCS combination at the ratio of 0.5 : 0.5 against OA-induced NAFLD might be mainly via lipogenesis inhibition and β-oxidation enhancement in the liver. The synergistic effects of EPA-PL and SCS make it possible to reduce the doses of EPA-PL or SCS to avoid side effects, which is of value for the development of dietary supplements or functional foods for preventing or treating NAFLD.

## 1. Introduction

Non-alcoholic fatty liver disease (NAFLD) is a type of fatty liver in the presence of hepatic steatosis and in the absence of alcohol consumption. NAFLD has been associated with insulin resistance and metabolic syndrome [1]. Orotic acid (OA), an intermediate in pyrimidine biosynthesis, is known to establish the model that can reflect the natural aetiologic setting of NAFLD [2]. At present, NAFLD is treated by bariatric surgery and drug intervention such as thiazolidinediones; however, these treatment approaches can lead to many side effects such as increase of weight and osteoporosis [3]. Therefore, it is necessary to find functional foods or dietary supplements to prevent or treat non-alcoholic fatty liver.

Sea cucumber is a conventional tonic in China, and it contains a wide variety of bioactive compounds such as polysaccharides, saponins and phospholipids [4]. Growing evidence shows that dietary sea cucumber could improve liver function [1,5]. According to our previous studies, eicosapentaenoic acid-enriched phospholipids (EPA-PLs) could mediate lipogenesis by downregulating hepatic sterol regulatory element-binding transcription factor 1 (SREBP-1c) and significantly increase fatty acid β-oxidation by activating hepatic peroxisome proliferator-activated receptor (PPARα) expression in diet-induced obese mice [6,7]. Sea cucumber saponin (SCS), as a kind of marine bioactive substance, has been proved to exhibit significant effects on alleviating insulin resistance [8], lowering lipid accumulation by enhancing β-oxidation via PPARα activation [9] and repressing lipase activity to increase fatty acid excretion in the faeces [10].

However, the raw materials of EPA-PL are scarce and EPA is readily oxidized [11]. More importantly, research studies have shown that excessive *n*-3 polyunsaturated fatty acids (PUFAs) could increase lipid peroxidation (LPO) [12]. In addition, several studies indicated that cytotoxic and haemolytic activities of SCS limit its application [13,14]. Therefore, it is urgent to find an alternative approach, which has a similar or even better effect at lower dose than either EPA-PL or SCS alone [15,16].

Theoretically, a combination of EPA-PL and SCS could show synergistic effect in lipid metabolism. However, there are no reports about synergistic effects of EPA-PL and SCS on OA-induced NAFLD. To evaluate the synergistic effects of EPA-PL and SCS on lipid metabolism in dietary OA-induced NAFLD rats, we measured the lipid content in the serum and liver, and the expression of crucial genes involved in hepatic fatty acid synthesis and β-oxidation.

## 2. Material and methods

### 2.1. Preparation of sea cucumber saponins and eicosapentaenoic acid-enriched phospholipids

The saponins were isolated from the sea cucumber *Pearsonothuria graeffei* by a previous procedure [9]. The powder was obtained by grinding air-dried body walls of *P. graeffei* and was then extracted four times with refluxing ethanol. The combined extracts were evaporated *in vacuo* and further partitioned between water and chloroform. The water layer was extracted with *n*-butanol and the organic layer was evaporated *in vacuo* to yield *n*-butanol extracts. Samples were applied on an HP20 resin column, and eluted with water, 80% ethanol and 100% ethanol in sequence. The fraction eluted with 80% ethanol was collected and evaporated to obtain SCS. The purity of the yielded SCS was 80.4% and the main ingredients were echinoside A and holothurin A according to our previous study [17].

The EPA-PL was prepared according to a previous method [18]. The fresh sea cucumber *Cucumaria frondosa* was purchased in the Nanshan aquatic market of Qingdao. The body walls of *C. frondosas* were ground into powder after vacuum freeze-drying. Then the powder was extracted by 20 times of the chloroform solution–methanol solution (2 : 1, v/v) overnight. The extracted solution was mixed with one-fourth of water after filtration. The mixture was placed into a separating funnel and maintained for 24 h and then the chloroform layer was collected and evaporated to dryness under vacuum, containing the total lipids. Then EPA-PL was separated from neutral lipids and glycolipids by silica-gel column chromatography using chloroform, acetone, chloroform–methanol (9 : 1, v/v), chloroform–methanol (2 : 1, v/v) and methanol sequentially as eluents. The chloroform–methanol (2 : 1, v/v) eluent and methanol eluent were collected and EPA-PL was obtained after the removal of organic solvent under vacuum. The purity of EPA-PL was confirmed to be 84.1% according to the method of Ames [19]. The fatty acid composition of EPA-PL was determined by an Agilent 6890 gas chromatograph with a flame-ionization detector. The column was a HPINNOW-AX capillary column (30 m × 0.32 mm × 0.25 µm). The temperature of the detector and injector was kept constant at 250 and 240°C, respectively, and the oven temperature was increased from 170 to 240°C at 3°C min^−1^ and held at 240°C for 15 min. Nitrogen was used as the carrier gas at a flow rate of 1.2 ml min^−1^. The main fatty acid composition of EPA-PL is shown in table 1.

**Table 1.** Main fatty acid compositions of EPA-PL (%).

fatty acid	EPA-PL
C16:0	5.23 ± 0.43
C16:1 *n*-9	1.59 ± 0.13
C17:0	0.92 ± 0.06
C18:2 *n*-6	2.60 ± 0.23
C18:3 *n*-6	1.74 ± 0.16
C20:0	1.77 ± 0.14
C20:3	14.9 ± 1.14
C20:5 *n*-3 (EPA)	59.7 ± 3.83
C22:6 *n*-3 (DHA)	1.51 ± 0.11

### 2.2. Animals and diets

All aspects of the experiment were conducted according to the guidelines provided by the ethical committee of experimental animal care at Ocean University of China (Qingdao, China). SD rats (Vital river, Beijing, China), weighing 240 ± 10 g at the beginning of the experiments, were acclimatized under a 12 L : 12 D cycle at 23°C and provided with food and water *ad libitum*. After a one-week adaptation period, the animals were divided into six groups consisting of seven rats each group. Rats were assigned to the following groups: (1) the normal control group (Con); (2) the orotic acid model group (OA); (3) the EPA-PL group; (4) the SCS group; (5) and (6) the combination of EPA-PL and SCS at half dose (EPA-PL : SCS, 0.5 : 0.5) and full dose (EPA-PL : SCS, 1 : 1) groups. The feed of each group was prepared on the basis of AIN93-M basic formula. The Con was supplied with an ordinary diet and the diets of the other five groups were prepared by supplementation of 1% orotic acid to the basal diet at the expense of corn starch as previously used. The EPA-PL group and SCS group were fed with 1% EPA-PL and 0.028% SCS, respectively. The ingredients of the six experimental diets are summarized in table 2. Body weight (BW) and food intake were recorded daily. After 10 days of feeding, rats were sacrificed after overnight fasting. The rats were sacrificed by blood extraction through the abdominal aorta after ether anaesthesia. Liver and white adipose tissues were quickly excised and their weights were determined. All the organs and tissues were frozen in liquid nitrogen and stored at −80°C until analysis. All protocols were approved by the Ethics College of Food Science and Engineering of Ocean University of China.

**Table 2.** Composition of experimental diets. ‘—’, none added.

ingredient composition (g kg^−1^)	Con	OA	EPA-PL	SCS	EPA-PL : SCS 0.5 : 0.5	EPA-PL : SCS 1 : 1
sucrose	100	100	100	100	100	100
casein	200	200	200	200	200	200
corn starch	499.5	489.5	487	489.2	488.1	486.7
corn oil	100	100	92.5	100	96.25	92.5
powdered cellulose	50	50	50	50	50	50
mineral mix	35	35	35	35	35	35
vitamin mix	10	10	10	10	10	10
dl-methionine	3	3	3	3	3	3
choline bitartrate	2.5	2.5	2.5	2.5	2.5	2.5
orotic acid	—	10	10	10	10	10
sea cucumber saponins	—	—	—	0.28	0.14	0.28
sea cucumber phospholipids	—	—	10	—	5	10

### 2.3. Analysis of hepatic lipids

Hepatic lipids were extracted and purified according to the method of Folch [20] and then dissolved with Triton X-100. The concentrations of triglycerides (TGs) and total cholesterol (TC) in the liver were measured using enzymatic reagent kits from Biosino (Beijing, China), and the hepatic phospholipid (PL) levels were determined according to a previous method [21].

### 2.4. Serum biochemistry analysis

Serum TG, TC, free fatty acid (FFA) and glucose levels, and aspartate aminotransferase (AST) and alanine aminotransferase (ALT) activities were measured using enzymatic reagent kits according to the manufacturer's instructions (Biosino, Beijing, China) [22].

### 2.5. RNA purification and quantitative real-time PCR

Real time-PCR (RT-PCR) was used to measure mRNA levels of related genes according to previous studies [23,24]. Total RNA was extracted from the liver using Trizol Reagent (Invitrogen, USA) [25]. Total RNA was reverse-transcribed into cDNA using random primer (TOYOBO, Japan). The purities of PCR products were assessed by the melt curve analysis. Relative gene expression was quantified using the standard curve method. Results were expressed as the relative values after normalization to glyceraldehyde 3-phosphate dehydrogenase (GAPDH). The primer sequences are provided in table 3.

**Table 3.** Primer sequences of RT-qPCR amplification.

gene	forward primer	reverse primer
FAS	TTGATGATTCAGGGAGTGGA	AGCAGATGAGTTGTTCTTGGAC
ME	TCACCTGCCCTAATGTCCCT	CATGCCGTTATCAACTTGTCC
G6PDH	GTTTGGCAGCGGCAACTAA	GGCATCACCCTGGTACAACTC
SREBP-1c	AACCTCATCCGCCACCTG	TGGTAGACAACAGCCGCATC
PPARα	GTACGGCAATGGCTTTATCA	CAATCCCCTCCTGCAACTT
ACOX1	GTATAAACTCTTCCCGCTCCTG	CCAGGTAGTAAAAGCCTTCAGC
GAPDH	TGATTCTACCCACGGCAAGTT	TGATGGGTTTCCCATTGATGA
ACC	CTAAACCAGCACTCCCGATT	ACTAGGTGCAAGCCAGACAT
CPT1	GCTTCCCCTTACTGGTTCC	AACTGGCAGGCAATGAGACT
CPT2	GCCCAAACCCCATTTTCTA	TAGGCAGAGGCAGAAGACAGCA

### 2.6. Statistical analysis

All results were expressed as mean ± s.e.m. (indicated by error bars). Statistical analyses were performed with SPSS18.0. Differences between the means were tested by one-way ANOVA, and all detected significant differences were further evaluated by Tukey's *post hoc* test. The level of significance chosen was *p* < 0.05.

## 3. Results and discussion

### 3.1. General observation

As described in table 4 (electronic supplementary material, data S1), the initial BW was similar between the six groups and there was no significant difference in food intake after treatment with samples for 10 days. The OA diet markedly decreased the BW gain in comparison with the control group, indicating that our model was created successfully. In addition, OA diet supplementation markedly elevated the epididymal adipose tissue weight and the liver weight by 22.9% (*p* < 0.05) and 43.0% (*p* < 0.01), respectively, while there was no significant difference in perirenal adipose tissue weight. This was consistent with the previous studies, which also showed the significant fatty liver of rats in the model of OA-induced NAFLD [1].

**Table 4.** Synergistic effect of EPA-PL and SCS on growth parameters in rats (*n* = 7). Date are presented as mean ± s.e.m.

	Con	OA	EPA-PL	SCS	EPA-PL : SCS 0.5 : 0.5	EPA-PL : SCS 1 : 1
initial body weight (g)	276.3 ± 4.4	276.4 ± 3.5^a^	276.2 ± 2.7^a^	276.4 ± 3.7^a^	276.3 ± 1.7^a^	276.3 ± 2.7^a^
body weight gain (g)	49.50 ± 3.5	40.43 ± 2.6*^a^	45.21 ± 3.7^a^	43.3 ± 2.7^a^	48.14 ± 2.5^b^	45.93 ± 2.3^a^
food intake (g d^−1^)	23.63 ± 0.4	23.80 ± 0.3^a^	23.87 ± 0.6^a^	24.25 ± 0.7^a^	23.97 ± 0.3^a^	23.5 ± 0.8^a^
organ weight (10 g kg^−1^ body weight)						
liver	3.05 ± 0.32	4.36 ± 0.17^**b^	4.00 ± 0.13^ab^	4.33 ± 0.20^b^	4.15 ± 0.13^ab^	3.59 ± 0.08^a^
White adipose tissue weight (10 g kg^−1^ body weight)						
epididymal	1.05 ± 0.06	1.29 ± 0.07^*b^	1.14 ± 0.05^a^	1.31 ± 0.05^b^	1.13 ± 0.04^a^	1.21 ± 0.05^ab^
perirenal	1.11 ± 0.16	1.13 ± 0.06^b^	0.89 ± 0.07^a^	1.21 ± 0.08^b^	0.94 ± 0.07^a^	1.06 ± 0.07^ab^

*p < 0.05, **p < 0.01 compared to the Con group. Different letters indicate significant difference at *p* < 0.05 determined by ANOVA (Tukey's test).

Interestingly, only the half dose combination of EPA-PL and SCS could increase the BW after treatment for 10 days, reaching a weight close to the Con group. The combination of EPA-PL and SCS at full dose noticeably downregulated the OA-induced liver weight by 17%, which showed excellent improvement. Additionally, EPA-PL and EPA-PL : SCS at the ratio of 0.5 : 0.5 also decreased liver weight to some degree, while there was no statistical difference. Importantly, the EPA-PL and the combination of EPA-PL and SCS at half dose significantly reduced the OA-induced weight increase in epididymal and perirenal tissue. Previous studies showed that *n*-3 PUFA-rich fish oil exhibited a positive effect on lowering the liver weight in high-cholesterol diet-induced NAFLD [26]. Overall, dietary supplementation with the combination of EPA-PL and SCS at half dose could improve the growth parameters of OA-induced NAFLD rats.

### 3.2. Serum parameters

NAFLD may be partly related to an increased risk of underlying metabolic abnormalities, such as glucose intolerance and dyslipidaemia [27]. We determined the changes in serum parameters including TG, TC, FFA and glucose in rats (table 5, electronic supplementary material, data S2). The present study showed that OA could significantly decrease both the serum TG and TC content (*p* < 0.001 and *p* < 0.05, respectively). A previous study also indicated that OA diet supplementation for 30 days could reduce the levels of serum TG and TC without significance [5]. However, the FFA and glucose levels were slightly lowered without significant difference after OA diet supplementation for 10 days in our present study. This might demonstrate that insulin resistance was not generated during the primary stages of fatty liver without hyperlipidaemia interference. Interestingly, our previous study found that OA diet supplementation for a long time (60 or 90 days) could lead to insulin resistance [5].

**Table 5.** Synergistic effect of EPA-PL and SCS on serum parameters in rats (*n* = 7). Date are presented as mean ± s.e.m.

serum parameters (mmol l^−1^)	Con	OA	EPA-PL	SCS	EPA-PL : SCS 0.5 : 0.5	EPA-PL : SCS 1 : 1
TG	0.78 ± 0.05	0.48 ± 0.10^*a^	0.67 ± 0.07^b^	0.51 ± 0.07^a^	0.63 ± 0.07^b^	0.57 ± 0.18^ab^
TC	2.66 ± 0.11	1.69 ± 0.10^***a^	2.32 ± 0.20^b^	1.99 ± 0.08^ab^	2.23 ± 0.06^b^	2.03 ± 0.14^b^
FFA	0.71 ± 0.08	0.61 ± 0.05^a^	0.66 ± 0.06^a^	0.73 ± 0.06^a^	0.67 ± 0.10^a^	0.77 ± 0.05^a^
glucose	8.28 ± 0.52	7.82 ± 0.36^a^	8.31 ± 0.37^a^	8.27 ± 0.30^a^	8.93 ± 0.29^b^	7.96 ± 0.26^a^

*p < 0.05, ***p < 0.001 compared to the Con group. Different letters indicate significant difference at *p* < 0.05 determined by ANOVA (Tukey's test).

EPA-PL treatment for 10 days could significantly increase both serum TG and TC levels in NAFLD rats fed with the OA diet. However, EPA-PL showed a positive effect on reducing serum TC and TG in diet-induced-obese mice [6,7]. The accumulation of serum lipids in NAFLD fed with EPA-PL might be attributed to reducing the accumulation of lipid in the liver and the enhancement of lipid efflux [28]. The combination of EPA-PL and SCS at half dose showed better effects than SCS alone and the combination of EPA-PL and SCS at full dose on increasing serum lipid levels of OA-induced rat, which was similar to EPA-PL. The glucose content in serum could be remarkably increased after treatment with the combination of EPA-PL and SCS at half dose for 10 days, which showed outstanding effect among all sample groups. The phenomenon might be attributed to the weakened abilities of the liver to use glucose [29]. The data indicated that the combination of EPA-PL and SCS at half dose showed advantages in improving serum lipids in the OA-induced NAFLD rats, which was consistent with the results of growth parameters.

### 3.3. Hepatic lipid profiling

**Figure 1.**
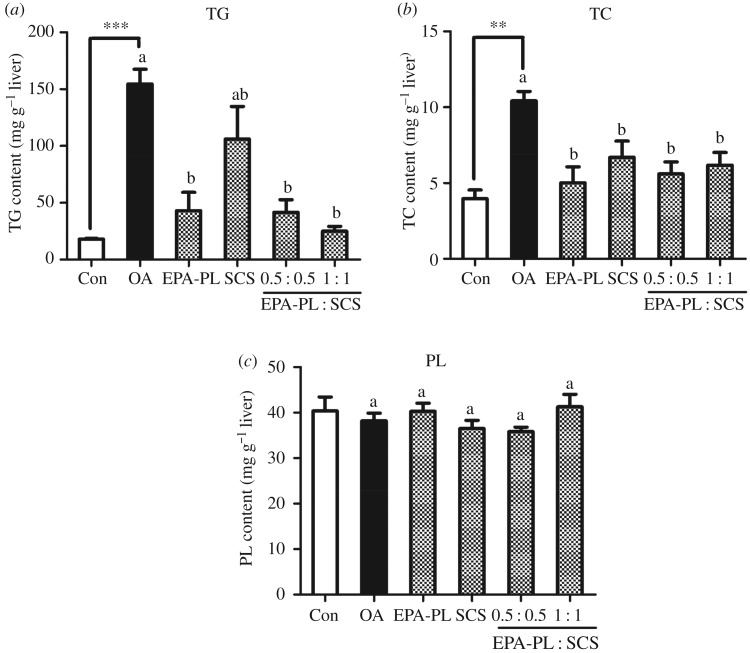
Synergistic effect of EPA-PL and SCS on hepatic lipid profiling in rats (*n* = 7). The levels of TG (*a*), TC (*b*) and PL (*c*) were measured from the rats' liver. Date are presented as mean ± s.e.m. (*n* = 7). **p < 0.01, ***p < 0.001 compared to the Con group. Different letters indicate significant difference at *p* < 0.05 determined by ANOVA (Tukey's test).

The liver plays a central role in lipid metabolism, which likely contributes to the onset and progression of chronic disease [30]. Orotic acid is known to cause lipid depositions in the liver over several days. This study was the first to explore the synergistic effect of EPA-PL and SCS on hepatic metabolism in OA-induced NAFLD. In the present study, we initially analysed the TC, TG and PL levels in the rat liver (figure 1, electronic supplementary material, data S3). Previous studies had testified that hepatic TG and TC accumulated in rats fed with OA as a result of stimulating lipogenesis [31] and impairing fatty acid catabolism [32]. Results indicated that OA intervention could significantly increase the contents of TG and TC in the liver instead of PL (figure 1, electronic supplementary material, data S3), manifesting that our model was created successfully, which was supported by the previous studies [9,33].

When the OA-induced rats were treated with EPA-PL alone, and the combination of EPA-PL and SCS at half or full dose for 10 days, the hepatic TG and TC contents were significantly reduced by different degrees. In another study, Liu *et al*. [4] also found EPA-PL from *C. frondosas* had protective effects against NAFLD [4]. However, SCS treatment for 10 days could only significantly reduce hepatic TC content, and the TG content was slightly decreased without remarkable difference.

The effects of samples on lowering hepatic lipids levels might be attributed to enhancing cholesterol excretion and improving the hepatic parameters of AST and ALT. Therefore, the AST and ALT levels were evaluated in the next experiment.

### 3.4. Hepatic parameters

**Figure 2.**
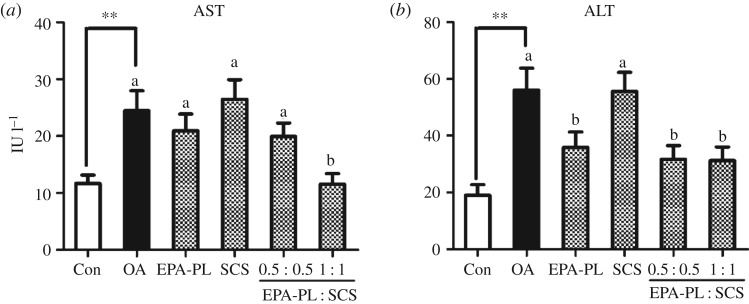
Synergistic effects of EPA-PL and SCS on hepatic parameters in OA-fed rats. The activities of AST (*a*) and ALT (*b*) were measured from the rats' serum. Date are presented as mean ± s.e.m. (*n* = 7). **p < 0.01 compared to the Con group. Different letters indicate significant difference at *p* < 0.05 determined by ANOVA (Tukey's test).

Serum AST and ALT levels as biochemical markers for liver health are commonly measured clinically. Figure 2 (electronic supplementary material, data S4) summarizes the hepatic enzyme activities of the rats after treatment with EPA-PL, SCS and their combinations for 10 days. Activities of AST and ALT in OA-induced NAFLD rats increased about onefold and twofold (*p* < 0.01), respectively, compared with the control group, indicating that rats fed with OA diet displayed severe NAFLD with liver injury. Those results were in agreement with our previous studies [4,34].

After the treatment with samples for 10 days, only the combination of EPA-PL and SCS at full dose could significantly decrease AST activity in OA-induced rats (figure 2*a*, electronic supplementary material, data S4). EPA-PL and the half dose combination of EPA-PL and SCS had the same effect on reducing AST content to some degree, but without significance in comparison with OA group (figure 2*a*, electronic supplementary material, data S4). Interestingly, EPA-PL and the combinations of EPA-PL and SCS at half or full dose all could dramatically decrease ALT activity in NAFLD rats (figure 2*b*, electronic supplementary material, data S4). Several studies have suggested that EPA is effective in reducing the serum AST and ALT activities [35,36]. However, SCS alone had no effect on reducing AST and ALT contents (figure 2, electronic supplementary material, data S4). This was consistent with our previous findings using 0.03% SCS rather than 0.1% SCS [1]. This result indicates that the different dosage of SCS might affect the activities of ALT and AST. The present study demonstrated that dietary supplementation of the combination of EPA-PL and SCS could markedly improve liver health.

### 3.5. Relative mRNA expressions of SREBP-1c and its relevant genes in the liver

**Figure 3.**
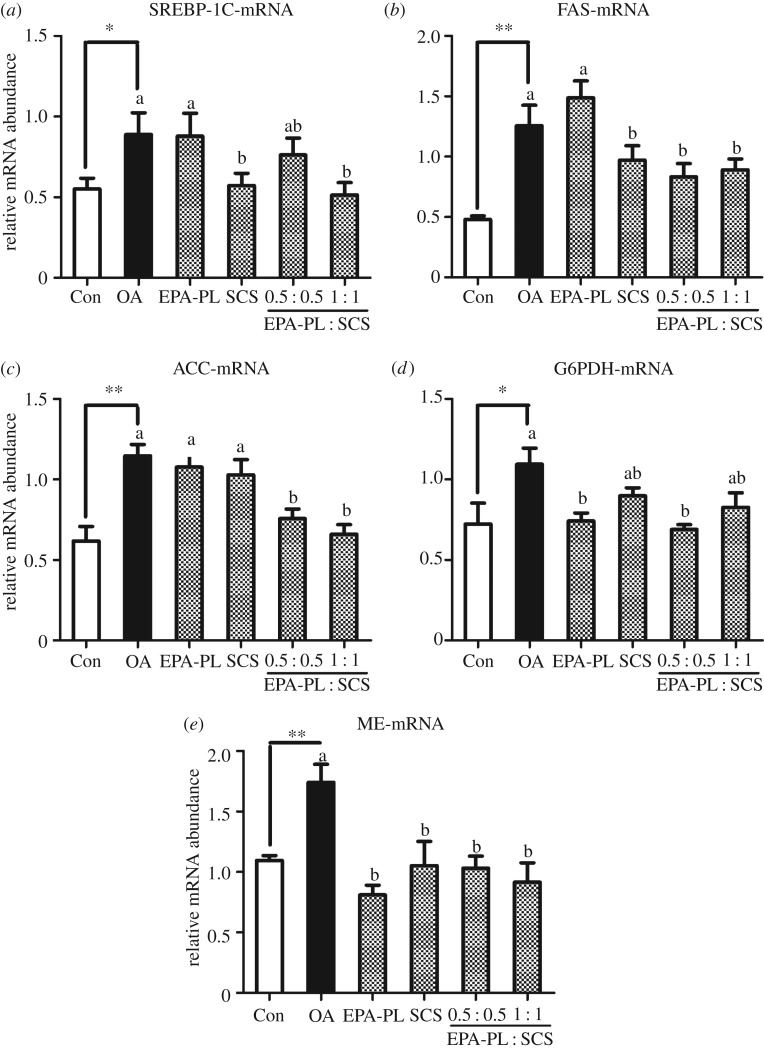
Synergistic effects of EPA-PL and SCS on hepatic mRNA expression involved in fatty acid biosynthesis in rats. The mRNA expression levels of SREBP-1c (*a*), FAS (*b*), ACC (*c*), G6PDH (*d*) and ME (*e*) were measured by RT-qPCR and results were normalized by GAPDH. Date are presented as mean ± s.e.m. (*n* = 7). *p < 0.05, **p < 0.01 compared to the Con group. Different letters indicate significant difference at *p* < 0.05 determined by ANOVA (Tukey's test).

The sterol-regulatory element-binding proteins (SREBPs) are central in the regulation of lipid biosynthetic pathways [37]. SREBP-1c (encoded by *SREBF1* gene) regulates the transcription of genes in lipid metabolism including fatty acid and TG metabolism [37]. The lipid accumulation in the liver of rats might potentially be caused by alterations in lipogenesis. Some key genes involved in fatty acid biosynthesis were measured in this study, such as SREBP-1c, fatty acid synthase (FAS), acetyl-CoA carboxylase (ACC), glucose-6-phosphate dehydrogenase (G6PDH) and malic enzyme (ME) (figure 3).

Results showed that OA administration markedly increased the mRNA expression of hepatic SREBP-1c (*p* < 0.05) and of the relevant genes including FAS, ACC, G6PDH and ME (figure 3). These results were accordant with previous studies [5,33], which further suggested our model was successful. Interestingly, the treatment of full dose combination of EPA-PL and SCS could significantly suppress the mRNA level of SREBP-1c, which nearly achieved the effect of the control group (figure 3*a*). Meanwhile, SCS showed an improvement similar to the combination of EPA-PL and SCS at half dose although there was no significant difference in comparison with the OA group. Importantly, the combination of EPA-PL and SCS at half dose could markedly reduce the expression levels of FAS, ACC, G6PDH and ME mRNA, which were very close to those of the control group (figure 3*b–e*). Moreover, the combination of EPA-PL and SCS at full dose significantly decreased the FAS, ACC and ME mRNA levels rather than that of G6PDH. The combination of EPA-PL and SCS inhibited the mRNA expression of ACC, FAS, ME and G6PDH by directly reducing SREBP-1c expression, which could result in the inhibition of hepatic endogenous fatty acid synthesis. Treatment with EPA-PL alone showed obvious downregulation only in G6PDH and ME mRNA levels, indicating that EPA-PL might decrease the liver lipids mainly through inhibiting the support of energy in fatty acid synthesis [33]. Meanwhile, SCS alone exhibited significant reduction only in FAS and ME mRNA expression, which was consistent with the previous study [9].

### 3.6. Relative mRNA expression of PPARα and its target genes in the liver

**Figure 4.**
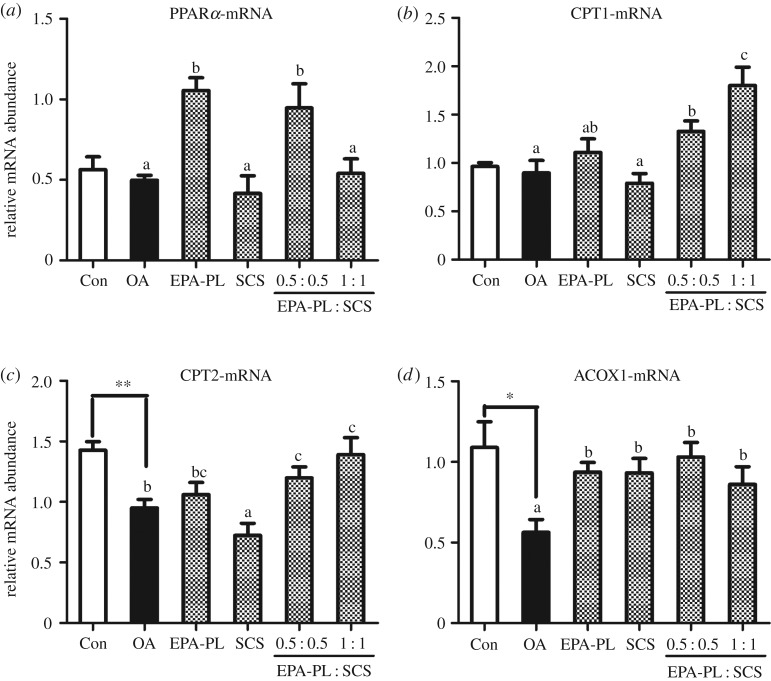
Synergistic effects of EPA-PL and SCS on hepatic mRNA expression involved in fatty acid *β*-oxidation in rats. The mRNA expression levels of PPARα (*a*), CPT1 (*b*), CPT2 (*c*) and ACOX1 (*d*) were measured by RT-qPCR and results were normalized by GAPDH. Date are presented as mean ± s.e.m. (*n* = 7). *p < 0.05 compared to the Con group. Different letters indicate significant difference at *p* < 0.05 determined by ANOVA (Tukey's test).

The accumulation of hepatic lipids is the result of decreased fatty acid oxidation. PPARα is a receptor protein with central roles in the modulation of lipid synthesis, transport and fatty acid metabolism [38,39]. Carnitine palmitoyl transferase 1 (CPT1) has crucial roles in the transport of fatty acids for β-oxidation, allowing the subsequent movement of acyl carnitine from the cytosol into the intermembrane space of mitochondria [38]. CPT2 is also an enzyme required for the acyl-carnitine shuttle that controls mitochondrial long-chain fatty acid oxidation [39]. β-Oxidation mainly occurs in both mitochondria and peroxisomes [30]. It is the catabolic process by which fatty acid molecules are broken down in the mitochondria in eukaryotes to generate acetyl-CoA, thereby entering the citric acid cycle [40]. Acyl-CoA oxidase 1 (ACOX1) is an important enzyme of the fatty acid β-oxidation pathway in the peroxisome, catalysing the desaturation of acyl-CoAs to 2-trans-enoyl-CoAs [41]. To examine the synergistic effect of EPA-PL and SCS on OA-induced NAFLD rats, we investigated the mRNA expression of PPARα and of its target genes including CPT1, CPT2 and ACOX1 in the liver (figure 4).

OA remarkably reduced the oxidation capacity, particularly downregulating the mRNA levels of CPT2 and ACOX1 (*p* < 0.01 and *p* < 0.05, respectively), which was in agreement with previous studies [40], further indicating that our model was created successfully. Interestingly, the combination of EPA-PL and SCS at half dose could significantly increase all of the genes related to fatty acid oxidation, including PPARα and its target genes (CPT1, CPT2 and ACOX1), improving both the transport and oxidation processes during fatty acid oxidation, which nearly matched those of the control group. The combination of EPA-PL and SCS at full dose markedly increased the mRNA expression of CPT1, CPT2 and ACOX1 instead of PPARα. The combination of EPA-PL and SCS at half dose exhibited better effects in upregulating mRNA expression of PPARα and its target genes in the liver, which might be attributed to the inverted U-shaped dose response [41]. In addition, EPA-PL alone could only significantly increase the PPARα and ACOX1 mRNA abundance. This was consistent with previous studies [33,42], which exposed that marine *n*-3 PUFAs could enhance fatty acid β-oxidation. However, the present study revealed that the SCS showed a converse effect on the mRNA expression of PPARα, CPT1 and CPT2, and it could significantly increase the ACOX1 mRNA level by 65%. These results were different from the previous study, which found the PPARα, CPT1, CPT2 and ACOX1 expression were significantly upregulated by 0.05% SCS treatment [9]. The different results might be attributed to the different dose of dietary SCS [1]. In general, the combination of EPA-PL and SCS at half dose exhibited significant improvement on fatty acid β-oxidation in OA-induced NAFLD rats.

**Figure 5.**
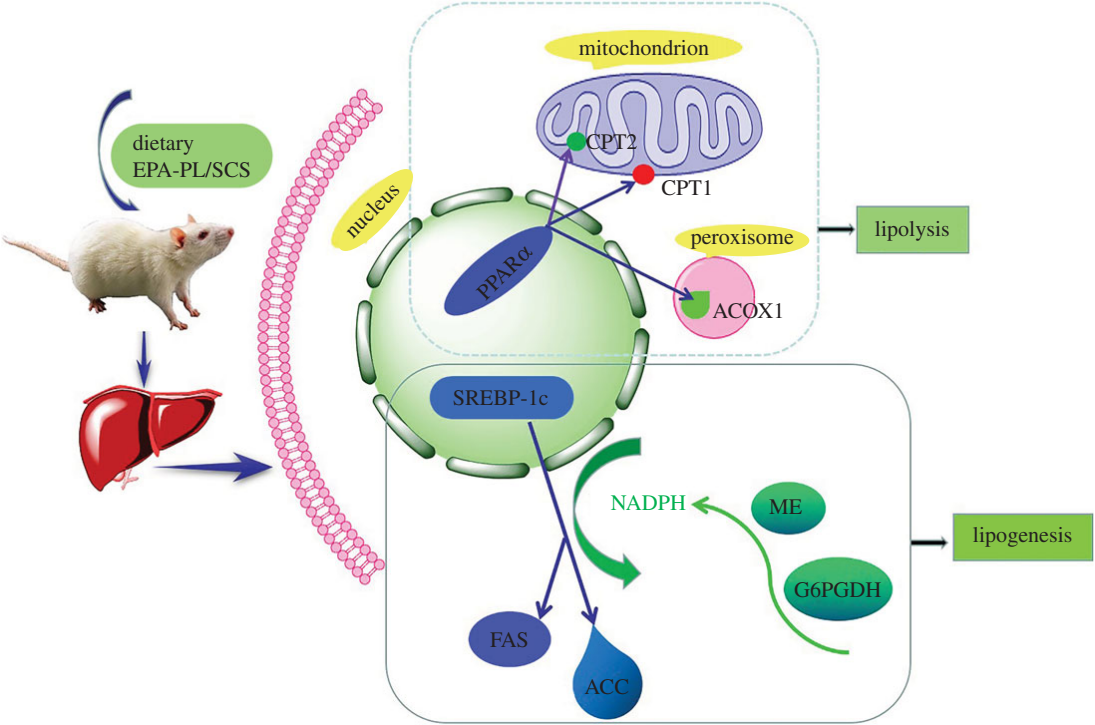
The possible mechanisms involved in the synergistic effects of EPA-PL and SCS in OA-induced NAFLD rats.

Overall, the combination of EPA-PL and SCS at half dose could significantly protect rats against OA-induced NAFLD mainly through inhibiting fatty acid synthesis and enhancing fatty acid β-oxidation (figure 5).

## 4. Conclusion

In conclusion, the present study evaluated the synergistic effect of EPA-PL and SCS on OA-induced NAFLD rats by determining growth parameters, serum parameters and liver function. Results showed that dietary supplementation of EPA-PL and SCS at half dose could markedly alleviate OA-induced symptoms. Further elucidation showed that the protective mechanism of the combination of EPA-PL and SCS at half dose against OA-induced NAFLD was mainly by inhibiting lipogenesis and enhancing fatty acid β-oxidation. The synergistic effects of EPA-PL and SCS make it possible to reduce the doses of EPA-PL or SCS to avoid side effects, which is of value for the development of dietary supplements or functional foods for preventing or treating NAFLD.

## Supplementary Material

Data S1

## Supplementary Material

Data S2

## Supplementary Material

Data S3

## Supplementary Material

Data S4

## Data accessibility

All relevant data are within the paper and the electronic supplementary material files.
